# A Case of Endometrial Stromal Sarcoma with Synchronous Bilateral Adenocarcinoma of Ovary

**DOI:** 10.1155/2012/687510

**Published:** 2012-11-06

**Authors:** Olga Caramelo, Carol Marinho, Teresa Rebelo, Natália Amaral, Fernando Mota, Fernanda Xavier da Cunha, Isabel Torgal

**Affiliations:** ^1^Gynecology and Obstetrics Department, Coimbra Hospital and University Center, Praceta Professor Mota Pinto, 3000-075 Coimbra, Portugal; ^2^Anatomical Pathology Department, Coimbra Hospital and University Center, Coimbra, Portugal

## Abstract

Endometrial stromal tumor is a rare mesenchymal uterine tumor. We report the case of a patient with endometrial stromal sarcoma and concomitant bilateral endometrioid adenocarcinoma of the ovary in the context of pelvic endometriosis. The patient underwent a complete cytoreduction including total hysterectomy and bilateral adnexectomy, pelvic lymphadenectomy, appendicectomy, infracolic omentectomy, and pelvic peritonectomy. This is the first report to our knowledge that describes a synchronous endometrial stromal sarcoma and bilateral endometrioid adenocarcinoma of the ovary.

## 1. Introduction

Endometrial stromal sarcoma (ESS) accounts for 0.2% of all malignant tumors of the female genital tract [1]. The mean age of women with ESS is 42–58 years; 10–25% of the women are premenopausal [1, 2].

Early on, endometrial stromal sarcoma (ESS) was subdivided into low grade and high grade categories mainly according to the mitotic count [1, 3].In current terminology, several pathologists suggested abandoning the term “high-grade endometrial stromal sarcoma” and using “undifferentiated or poorly differentiated endometrial sarcoma” to designate this malignancy [4, 5]. The term ESS is reserved for the previously known “low grade ESS”.

Various hypothetical risk factors observed for ESS are pelvic irradiation and long-term tamoxifen therapy [6]. Sporadic ESS could arise from pelvic endometriosis, namely, extraovarian endometriosis [7].

Endometriosis is a common benign, estrogen dependent, chronic gynecological disorder associated with pelvic pain and infertility. It is characterized by the presence and growth of endometrial glands and stromal outside the uterine cavity [8–10]. 

Prior studies have found an increased overall cancer incidence in women with endometriosis [11, 12] and support a positive association between ovarian endometriomas and ovarian cancer risk [13].

Despite being benign, the endometrium in endometriosis behaves like a tumoral tissue expression of growth factors involved in tumor proliferation, invasiveness, angiogenesis, lack of response to the mechanisms of normal regulation, susceptibility of relapse after complete excision, and ability to invade adjacent tissue and distant locations all being documented in endometriosis [9, 10, 14, 15].

Endometriosis has mixed traits of benignity and malignancy. The pathogenesis involves loss of control of cell proliferation and it is associated with local and distant spread. Although endometriosis cannot be termed a premalignant condition, epidemiologic, histopathologic, and molecular data suggest that endometriosis has a malignant potential [14].

Unlike typical ovarian cancers, those that arise from endometriosis have discrete features: they are more commonly constituted by clear cell and endometrioid subtypes, tend to be found in earlier stages, and have a favorable prognosis. Due to these unique characteristics, ovarian cancers derived from the malignant transformation of endometriosis have been specifically referred to as endometriosis-associated ovarian cancer (EAOC) [16–18].

We present a rare case involving a patient with endometrial stromal sarcoma and concomitant bilateral endometrioid adenocarcinoma of the ovary.

## 2. Case Presentation

A 43-year-old patient was sent from another institution presenting an adnexal mass and elevated tumor markers: cancer antigen 125 (CA 125)-5232 IU/mL; CA 19.9-468 IU/mL; CA 15.3-910 IU/mL IU/mL, CEA-6.9 ng/mL; alpha-fetoprotein 1.9 ng/mL. Her relevant medical history included epilepsy and two recent previous episodes of deep venous thromboembolism. She was a gravid 3 para 1, with two spontaneous first trimester miscarriages. No previous surgeries were known.

**Figure 1:**
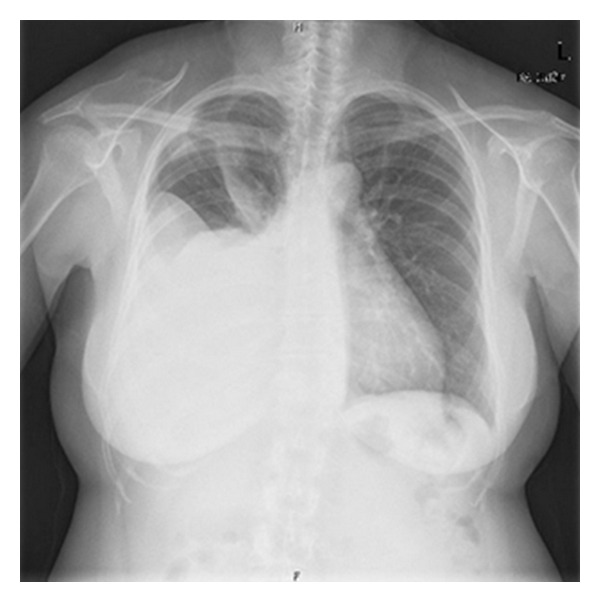
Chest scan with a large pleural effusion.

The abdominal exam showed a hypogastric pain and a hard-to-define mass. Gynecological exam revealed a hard-to-delimitate uterus, a painful posterior vaginal wall, and enlarged adnexal regions. Vaginal ultrasound was performed and revealed a slight uterine enlargement with an image of a nodular interstitial formation measuring 32 × 31 mm, an enlarged right ovary (84 × 62 mm) containing a cystic image with heterogeneous filling, and an enlarged left ovary also with central cavitation. The chest scans showed a large pleural effusion (Figure 1).

**Figure 2:**
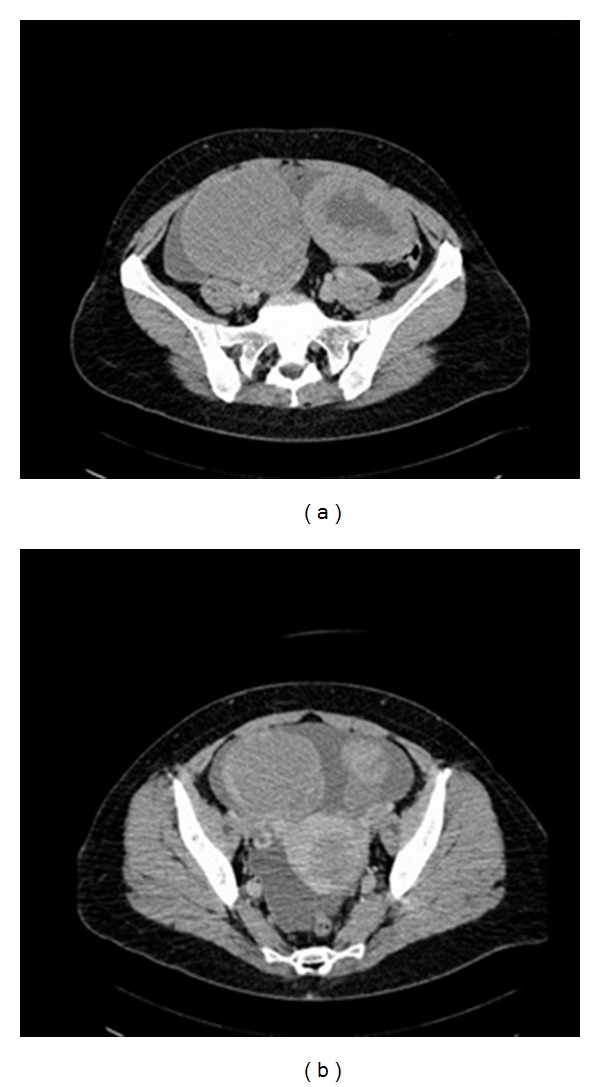
Computed tomography (CT) showed (a) a partially necrotic solid mass measuring 9.5 cm in diameter in the left side of the pelvis and a cystic tumor in right side measuring 10.5 cm. (b) The uterus presented an heterogeneous myometrial structure with a nodular lesion 4 cm long.

The patient was submitted to thoracentesis (with drainage of 1500 cc of fluid) and the cytological analysis revealed inflammatory exudates without neoplastic cells. Esophagoduodenoscopy and colonoscopy revealed no abnormalities. Pap smear, mammography, and breast ultrasound were normal. Abdominopelvic computed tomography (CT) (Figure 2) showed a partially necrotic solid mass measuring 9.5 cm in diameter in the left side of the pelvis and a cystic tumor in right side measuring 10.5 cm. The uterus presented a heterogeneous myometrial structure with a 4-cm-nodular lesion. There was densification of omental fat suggesting possible metastasis and moderate ascites.

The patient underwent total hysterectomy and bilateral adnexectomy, pelvic lymphadenectomy, appendicectomy, infra-colic omentectomy, and pelvic peritonectomy. During the laparotomy, the patient was transfused with two units of packed red blood cells. Both ovaries were transformed to solid tumor masses 10 cm long, there were peritoneal implants less than 1 cm scattered throughout the pelvic excavation and epiplon, and the uterus was enlarged and hard. The postoperative period was uneventful, and the patient was discharged on the fifth day without complications.

The pathology report stated that the left ovary was transformed to a cystic formation; it weighed 350 g and measured 12 × 10 × 10 cm. The external surface was lobulated and predominantly bright pink. There was a tumoral-looking area of infiltration of the capsule extending along 1 cm. Upon sectioning, there were areas of friable edematous aspect.

 The right ovary was transformed into a cystic formation with no content coated with a smooth surface. The internal surface showed multiple vegetations of yellow softened tissue measuring 0.2 to 2 cm.

Microscopically the left ovary exhibited clumps of cells, architecturally of cribiform endometrioid type. Tumoral cells had hyperchromatic nuclei with moderate atypia, with solid areas where there was a more pronounced pleomorphism. The tumor infiltrated adjacent ovarian parenchyma with ovarian capsule disruption. Microscopically, the right ovary presented endometrioid glandular structures with moderate pleomorphism, hyperchromatic nuclei, and an increased nucleus/cytoplasm ratio. On the surface, there were solid areas with cells featuring marked nuclear pleomorphism. In the transition to the nontumoral epithelial tissue, there was an endometrial-type epithelium with little underlying stromal and reactive modifications.

The uterus weighed 200 g and measured 9 cm in height, presented a fasciculate myometrium with a thickness ranging from 1 to 3 cm: in its posterior wall, a 4-cm hard nodular structure with ill-defined limits was identified. Microscopically the nodular formation exhibited a high cellular density and a solid pattern. The large cells presented a high nucleus/cytoplasm ratio. The nucleus showed slight atypia. The pattern of growth was mainly expansive, infiltrating the adjacent myometrium with vascular embolization. 

The immunohistochemical features found in the histology specimen of the uterus included reactivity to antibodies specific for vimentin and negativity for epithelial markers cytokeratin (CK7) and pankeratin MNF116, smooth muscle actin, desmin, HMB45, CD68, and calponin. The presence of vascular embolization was confirmed through positive immunoreactivity to factor VIII and CD34. Estrogen, progesterone receptors, and Bcl2 were positive. Immunoreactivity to CD10 was not found, and external advice was sought which confirmed the negative reaction to CD10. The index of cell proliferation (Ki67) was 10%.

**Figure 3:**
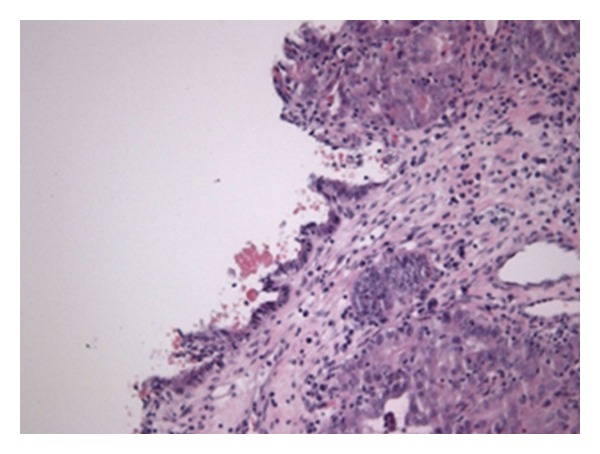
Endometrioid adenocarcinoma of the ovary, moderately differentiated, showing aspects that suggest an endometriosis origin.

**Figure 4:**
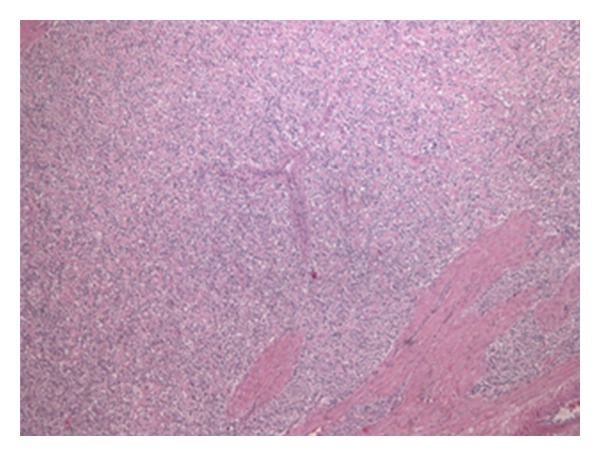
Endometrial stromal sarcoma, detail of stromal cells (50X).

The pathologic study revealed bilateral endometrioid adenocarcinoma of the ovary, moderately differentiated (Figure 3), showing aspects that suggested origin in an endometriosis cyst in a context of known pelvic endometriosis and disruption of the left ovarian capsule. It was classified in the FIGO stage IIC. The uterus showed a 6-cm intra-myometrial mesenchymal neoplasm on the posterior wall of the uterine body, an endometrial stromal sarcoma (Figure 4) with intravascular extension, and monotonous collections of tumor cells with low mitotic activity. This tumor was classified as stage IC. There was no metastatic disease in all 13 pelvic nodes excised. No disease was found in the ileocecal appendix, epiploon, or peritoneum. No residual disease was found in this context.

The patient subsequently underwent chemotherapy with six cycles of paclitaxel and carboplatin. A clinical suspicion of a vaginal fistula prevented treatment with vaginal brachytherapy (28 Gy/4F). A clinical follow-up at six months and a CT scan 1 year after surgery revealed no adenopathy or peritoneal effusion; the value of CA 125 was 19 IU/mL. Currently, the patient is under hormonal therapy with tamoxifen.

## 3. Discussion

To the best of our knowledge, this is the first report that describes a synchronous ESS and bilateral endometrioid adenocarcinoma of the ovary. Several studies have documented synchronous bilateral ovary and endometrial cancer but until now none reported a synchronous ESS.

The frequency of malignant transformation of endometriosis is unknown, but it is estimated that up to 1% of women with endometriosis will develop an endometriosis-associated neoplasm [1, 2, 7, 9, 11, 19–21]. Common pathogenetic factors of both endometriosis and ovarian malignancy include familial predisposition, genetic alterations, cell adhesion, and immunobiologic, angiogenic and hormonal factors [14]. The demonstration of a dysplastic phase between the benign endometriosis and the carcinoma is a criterion for the diagnosis of neoplastic transformation [1, 2, 7, 9, 11, 19–21]. The pathological analysis of the present case demonstrated such histological transition. 

Several laboratory and clinical data suggest that ESS are hormonally sensitive [19, 21, 22]. The estrogen (ER) and progesterone receptor (PR) expression may have implications for hormone therapy in the management of these tumors, suggesting that ER and PR should be routinely quantified in ESS by immunohistochemical methods [23].

Although CD10 immunoreactivity is a well-known positive predictive marker of ESS, there are exceptional cases when CD10 is negative, namely, in fibrous variants [24].

Surgery has always been described as the most effective treatment for uterine sarcomas. Total abdominal hysterectomy with bilateral salpingo-oophorectomy is considered to be the standard treatment for ESS [3, 21, 25, 26]. Some data on small series reported a nodal involvement in 33–45% of the patients undergoing lymph node dissection during primary or secondary surgical treatment, thus suggesting a role for lymphadenectomy in this malignancy [27–29]. Nevertheless, the potential prognostic significance of the presence of lymph node metastases in low-grade endometrial stromal sarcoma is still unknown [29].

The options of adjuvant therapy following surgery include radiotherapy, chemotherapy, and hormonal therapy [20, 30]. Adjuvant radiotherapy appears to improve local control without any significant impact on overall survival [29, 31]. The high recurrence rate and the metastatic tendency make these tumors good candidates for systemic therapy which was prescribed in the present case. The risk of recurrence is thought to be as high as 50%, although these tumors are usually slow growing and recurrences occur later [32, 33]. 

Prolonged survival and even cure are common after surgical resection of recurrent or metastatic lesions [34].

These tumors have usually an indolent clinical course with 80–100% 5-year survival, but about 37–60% of patients eventually recur after a very long time and 15–25% die of the disease [26, 35]. Prognostic factors in patients with ESS are still controversially discussed [3, 36]. Early tumor stage, low myometrial invasion, and low mitotic count were shown as prognostic factors associated with a lengthened overall survival. On the contrary, age, histologic grade, and adjuvant therapy showed no influence on the overall survival of patients with ESS [36].

Patients with a uterine sarcoma should be treated within well-designed, randomized clinical trials, that are difficult to be conducted because of the rarity of such malignancies [29]. It is plausible that in this case both tumors originated from the same pathologic entity which is ovarian endometriosis and uterine adenomyosis. The association of both tumors could be related to the foci of malignant transformation of endometriosis/adenomyosis [2]. In conclusion, we present a very rare pathological entity occurring as a malignant disease, possibly related to a previous but not reported endometriosis, and that consisted of a bilateral endometrioid adenocarcinoma of the ovary and a synchronous EES of the uterus.

## References

[1] De Fusco PA, Gaffey TA, Malkasian GD, Long HJ, Cha SS (1989). Endometrial stromal sarcoma: review of Mayo Clinic experience, 1945–1980. *Gynecologic Oncology*.

[2] Chang KL, Crabtree GS, Lim-Tan SK, Kempson RL, Hendrickson MR (1990). Primary uterine endometrial stromal neoplasms. A clinicopathologic study of 117 cases. *American Journal of Surgical Pathology*.

[3] Gadducci A, Sartori E, Landoni F (1996). Endometrial stromal sarcoma: analysis of treatment failures and survival. *Gynecologic Oncology*.

[4] Amant F, Vergote I, Moerman P (2004). Response: the classification of a uterine sarcoma as ’High-grade endometrial stromal sarcoma’ should be abandoned. *Gynecologic Oncology*.

[5] Moinfar F, Azodi M, Tavassoli FA (2007). Uterine sarcomas. *Pathology*.

[6] Yildirim Y, Inal MM, Sanci M (2005). Development of uterine sarcoma after tamoxifen treatment for breast cancer: report of four cases. *International Journal of Gynecological Cancer*.

[7] Benoit L, Arnould L, Cheynel N (2006). Malignant extraovarian endometriosis: a review. *European Journal of Surgical Oncology*.

[8] Farquhar CM (2000). Extracts from “Clinical Evidence”: endometriosis. *British Medical Journal*.

[9] Bulun SE (2009). Endometriosis. *New England Journal of Medicine*.

[10] Giudice LC, Kao LC (2004). Endometriosis. *The Lancet*.

[11] Brinton LA, Lamb EJ, Moghissi KS (2004). Ovarian cancer risk associated with varying causes of infertility. *Fertility and Sterility*.

[12] Borgfeldt C, Andolf E (2004). Cancer risk after hospital discharge diagnosis of benign ovarian cysts and endometriosis. *Acta Obstetricia et Gynecologica Scandinavica*.

[13] Kobayashi H (2009). Ovarian cancer in endometriosis: epidemiology, natural history, and clinical diagnosis. *International Journal of Clinical Oncology*.

[14] Nezhat F, Datta MS, Hanson V, Pejovic T, Nezhat C, Nezhat C (2008). The relationship of endometriosis and ovarian malignancy: a review. *Fertility and Sterility*.

[15] Fritel X (2007). Endometriosis anatomoclinical entities. *Journal de Gynecologie Obstetrique et Biologie de la Reproduction*.

[16] Eržen M, Rakar S, Klančar B, Syrjänen K (2001). Endometriosis-associated ovarian carcinoma (EAOC): an entity distinct from other ovarian carcinomas as suggested by a nested case-control study. *Gynecologic Oncology*.

[17] Van Gorp T, Amant F, Neven P, Vergote I, Moerman P (2004). Endometriosis and the development of malignant tumours of the pelvis. A review of literature. *Best Practice and Research*.

[18] Mandai M, Yamaguchi K, Matsumura N, Baba T, Konishi I (2009). Ovarian cancer in endometriosis: molecular biology, pathology, and clinical management. *International Journal of Clinical Oncology*.

[19] Pink D, Lindner T, Mrozek A (2006). Harm or benefit of hormonal treatment in metastatic low-grade endometrial stromal sarcoma: single center experience with 10 cases and review of the literature. *Gynecologic Oncology*.

[20] Burke C, Hickey K (2004). Treatment of endometrial stromal sarcoma with a gonadotropin-releasing hormone analogue.. *Obstetrics and gynecology*.

[21] Chu MC, Mor G, Lim C, Zheng W, Parkash V, Schwartz PE (2003). Low-grade endometrial stromal sarcoma: hormonal aspects. *Gynecologic Oncology*.

[22] Reich O, Regauer S, Urdl W, Lahousen M, Winter R (2000). Expression of oestrogen and progesterone receptors in low-grade endometrial stromal sarcomas. *British Journal of Cancer*.

[23] Garrett A, Quinn MA (2008). Hormonal therapies and gynaecological cancers. *Best Practice and Research in Clinical Obstetrics and Gynaecology*.

[24] McCluggage WG (2007). Immunohistochemistry as a diagnostic aid in cervical pathology. *Pathology*.

[25] Li AJ, Giuntoli RL, Drake R (2005). Ovarian preservation in stage I low-grade endometrial stromal sarcomas. *Obstetrics and Gynecology*.

[26] Berchuck A, Rubin SC, Hoskins WJ, Saigo PE, Pierce VK, Lewis JL (1990). Treatment of endometrial stromal tumors. *Gynecologic Oncology*.

[27] Riopel J, Plante M, Renaud MC, Roy M, Têtu B (2005). Lymph node metastases in low-grade endometrial stromal sarcoma. *Gynecologic Oncology*.

[28] Reich O, Winter R, Regauer S (2005). Should lymphadenectomy be performed in patients with endometrial stromal sarcoma? [2] (multiple letters). *Gynecologic Oncology*.

[29] Gadducci A, Cosio S, Romanini A, Genazzani AR (2008). The management of patients with uterine sarcoma: a debated clinical challenge. *Critical Reviews in Oncology/Hematology*.

[30] Leunen M, Breugelmans M, De Sutter P, Bourgain C, Amy JJ (2004). Low-grade endometrial stromal sarcoma treated with the aromatase inhibitor letrozole. *Gynecologic Oncology*.

[31] Dusenbery KE, Potish RA, Judson P (2004). Limitations of adjuvant radiotherapy for uterine sarcomas spread beyond the uterus. *Gynecologic Oncology*.

[32] Schilder JM, Hurd WW, Roth LM, Sutton GP (1999). Hormonal treatment of an endometrial stromal nodule followed by local excision. *Obstetrics and Gynecology*.

[33] Fekete PS, Vellios F (1984). The clinical and histologic spectrum of endometrial stromal neoplasms: a report of 41 cases. *International Journal of Gynecological Pathology*.

[34] Livi L, Paiar F, Shah N (2003). Uterine sarcoma: twenty-seven years of experience. *International Journal of Radiation Oncology Biology Physics*.

[35] Inayama Y, Shoji A, Odagiri S (2000). Detection of pulmonary metastasis of low-grade endometrial stromal sarcoma 25 years after hysterectomy. *Pathology Research and Practice*.

[36] Bodner K, Bodner-Adler B, Obermair A (2001). Prognostic parameters in endometrial stromal sarcoma: a clinicopathologic study in 31 patients. *Gynecologic Oncology*.

